# Clinical, pathological, and molecular data concerning *Coenurus cerebralis* in Sheep in Egypt

**DOI:** 10.1016/j.dib.2017.10.070

**Published:** 2017-11-04

**Authors:** Said Amer, Ahmed ElKhatam, Yasuhiro Fukuda, Lamia I. Bakr, Shereif Zidan, Ahmed Elsify, Mostafa A. Mohamed, Chika Tada, Yutaka Nakai

**Affiliations:** a Department of Zoology, Faculty of Science, Kafr El Sheikh University, Kafr El Sheikh 33516, Egypt; b Department of Parasitology, Faculty of Veterinary Medicine, University of Sadat City University, Menofia, Egypt; c Laboratory of Sustainable Environmental Biology, Graduate School of Agricultural Science, Tohoku University, 232-3 Yomogida, Naruko-onsen, Osaki, Miyagi 989-6711, Japan; d Department of Zoology, Faculty of Science, Tanta University, El Gharbya, Egypt; e Department of Animal Hygiene and Zoonoses, Faculty of Veterinary Medicine, University of Sadat City, Menofia, Egypt; f Department of Animal Medicine and Infectious Diseases, Faculty of Veterinary Medicine, University of Sadat City, Menofia 32897, Egypt; g Department of pathology, Faculty of Veterinary Medicine, Menofia University, Menofia, , Egypt

**Keywords:** *Taenia multiceps*, *Coenurus cerebralis*, Sheep, Pathology, 12S rRNA, Egypt

## Abstract

This article contains information related to a recent study “Prevalence and Identity of *Taenia multiceps* cysts “*Coenurus cerebralis*” in Sheep in Egypt” (Amer et al., 2017) [1]. Specifically, affected sheep showed neurological disorders manifested as depression, head shaking and circling, altered head position, incoordination and paralysis in some cases. Brain-derived cysts were molecularly identified by PCR-sequence analysis at mitochondrial 12S rRNA gene marker. Cyst-induced pathological changes included degenerative changes and demyelination in brain tissue, infiltration of lymphocytes and histiocytes. Cystic fluids were biochemically analyzed for protein, lipids and electrolytes. The data of this study provides more understanding on phylogeny, epidemiology and pathology of coenurosis in sheep.

**Specifications Table**

**Table:** 

Subject area	Biology
More specific subject area	Parasitology
Type of data	Table, image (clinical cases in sheep, microscopy, etc.), text file and figure
How data was acquired	Clinical examination, microscopy, PCR-Sequence data, phylogenetic analysis.
Data format	Raw and analyzed data.
Data source location	El Menoufia Province (90 km South-East of Cairo), Egypt
Data accessibility	Data provided in the article is accessible to the public
Related research article	“Prevalence and Identity of *Taenia multiceps* cysts “*Coenurus cerebralis*” in Sheep in Egypt”

**Value of the Data**

• This work creates a deeper understanding of the epidemiology of *Coenurus cerebralis* in sheep.
• Data in this work provide more details on phylogeny of *Taenia multiceps*.
• Data in this work provide further understanding on pathological effects of *Coenurus cerebralis* on brain tissue of sheep.

## 1 Data

*Coenurus cerebralis* is the larval stage of the canide cestode *Taenia multiceps*. The predilection niche of coenurus cysts is the brain and spinal cord of sheep, as an intermediate host, resulting in neurological disorders. Infection rate in Egypt was about 11% based on clinical signs and 3% based on postmortem investigation of heads of slaughtered sheep [1]. Due to the substantial incidence in the Mediterranean basin, it needs more cooperation between researches from related countries to understand the epidemiology of this neglected disease and to propose the intervention measures to control it. We propose to establish a regional/global network for this purpose.

### 1.1 Clinical signs

**Fig. 1:**
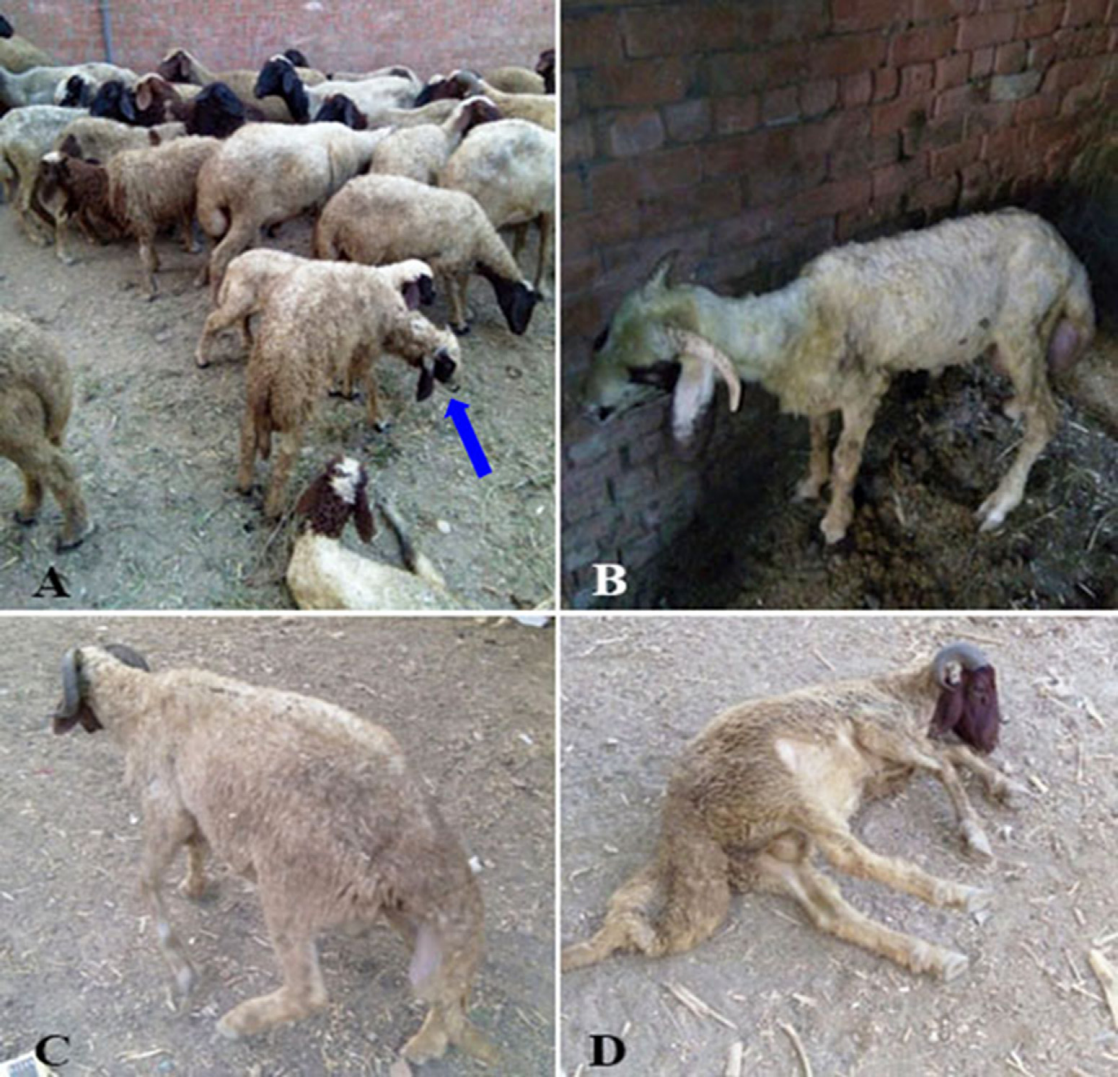
Photos of sheep showing different clinical signs.

Clinical signs of infected sheep included lateral deviation of the head and circling movement (Fig. 1A), head shaking, unilateral blindness, head pressing against the wall and tendency to keep away from other animals in the flock (Fig. 1B), depression, incoordination and ataxia (Fig. 1C) and lateral recumbancy with stretched limbs and convulsions (Fig. 1D). These signs are not differential diagnosis of coenursis in sheep because it is largely similar to that resulting from brain abscesses and infection with larvae of *Oesterus ovis*. Postmortem examination of the slaughtered animals is the gold standard for confirmation due to poor prognosis of infected cases and unaffordability of recent technology such as Computed Tomography (CT).

### 1.2 Coenurus cysts

**Fig. 2:**
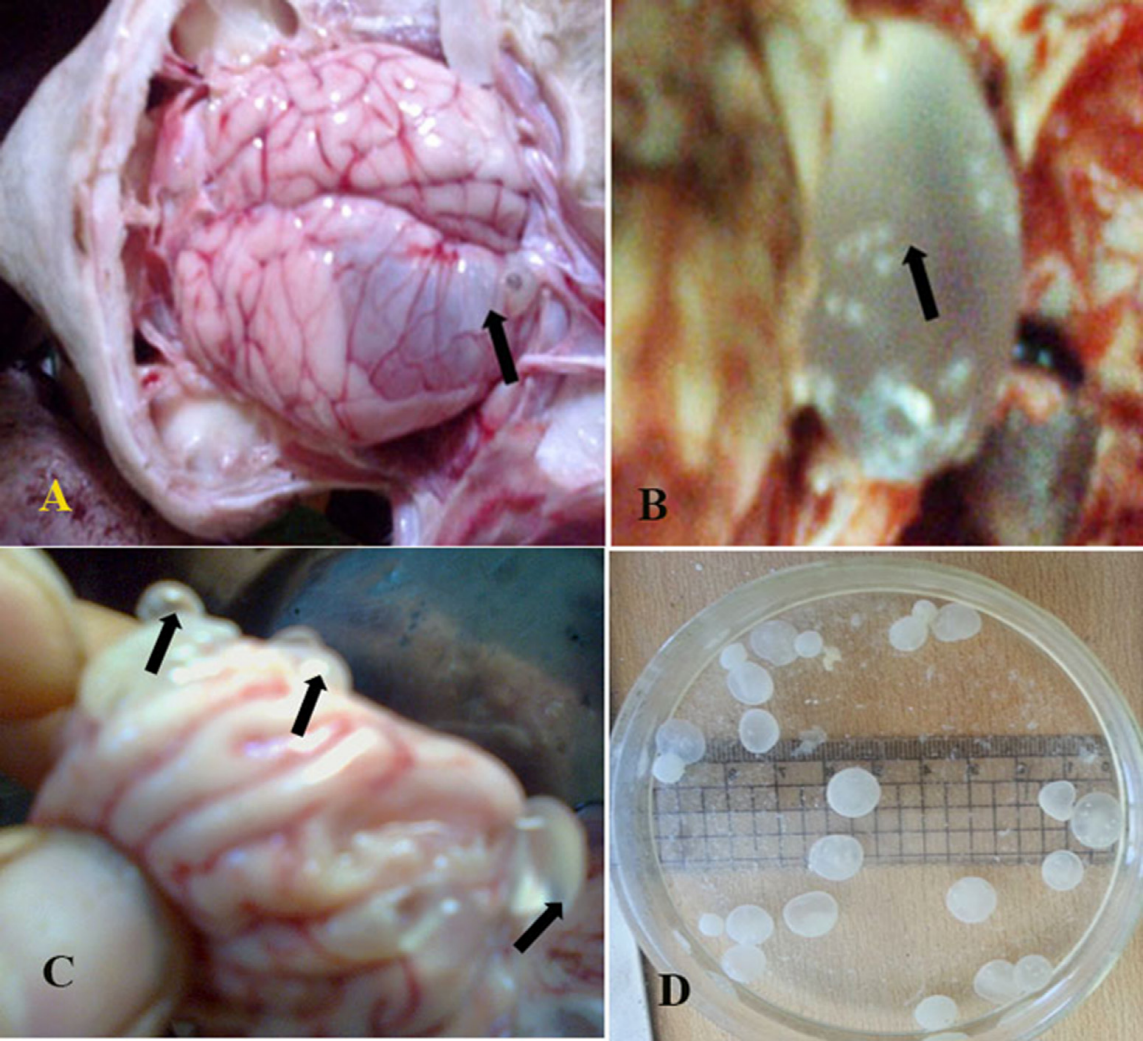
Photos of sheep showing coenurus cysts localized in the brain of slaughtered sheep and detached cysts.

**Fig. 3:**
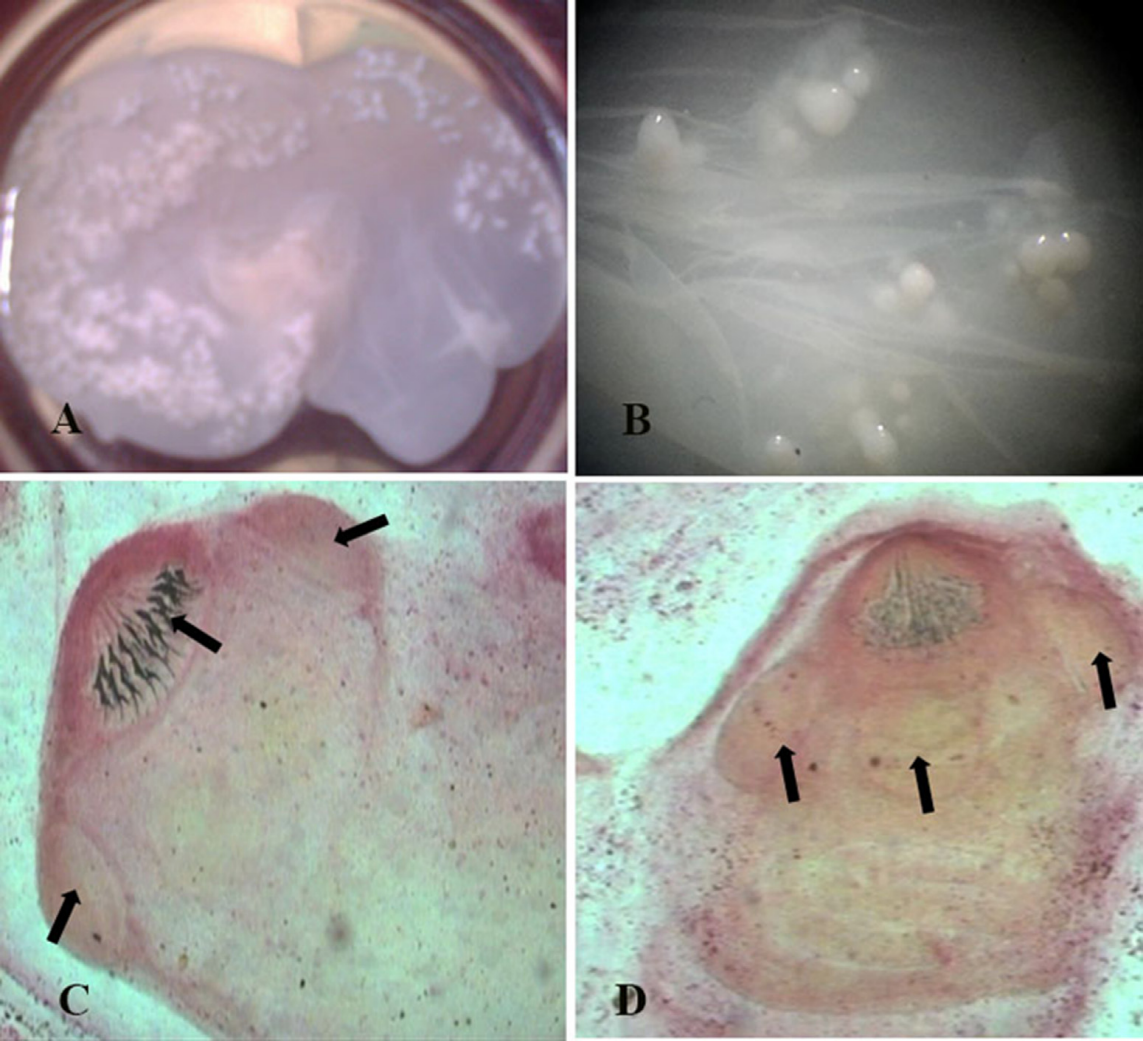
Photos of sheep showing coenurus cysts with visible protoscolices (A and B) and mounted scolex (C and D) with rostellum, hooks and suckers.

Recovered coenurii from infected brains were bladder-like cysts measuring 1.0×1.5 cm–4.5×7.0 cm in diameter (Figs. 2A, B and D and 3A). Some cases had one cyst (Fig. 2A and B) and some other cases had multiple cysts (Fig. 2C). Single cyst in each brain was found in about 90% of infected sheep while, double cysts were found in about 10% of infected cases. Only one sheep was infected with about 40 small cysts that distributed in all parts of brain (cerebrum, cerebellum and brain stem). Cysts composed of hyaline membrane with many protoscolices on the inner surface and filled with a translucent fluid of varying volume (Figs. 2B and 3A and B). The scolices per cyst varied in number from 5 scolices in the small one to about 400 scolices in the large one (Figs. 2B and 3A). The size individual scolex ranged from 0.45×0.5 to 0.50×0.65 mm with average size of 0.475×0.575 mm. Each Scolex consisted of 4 suckers and rostellum (Fig. 3C and D). The sucker size ranged from 0.14×0.18 to 0.15×0.20 mm (average 0.145×0.19 mm) in diameter. The rostellum of each scolex was armed with 18–28 (average 23) hooks arranged in two rows of large and small hooks (Fig. 3C).

### 1.3 Pathological data

**Fig. 4:**
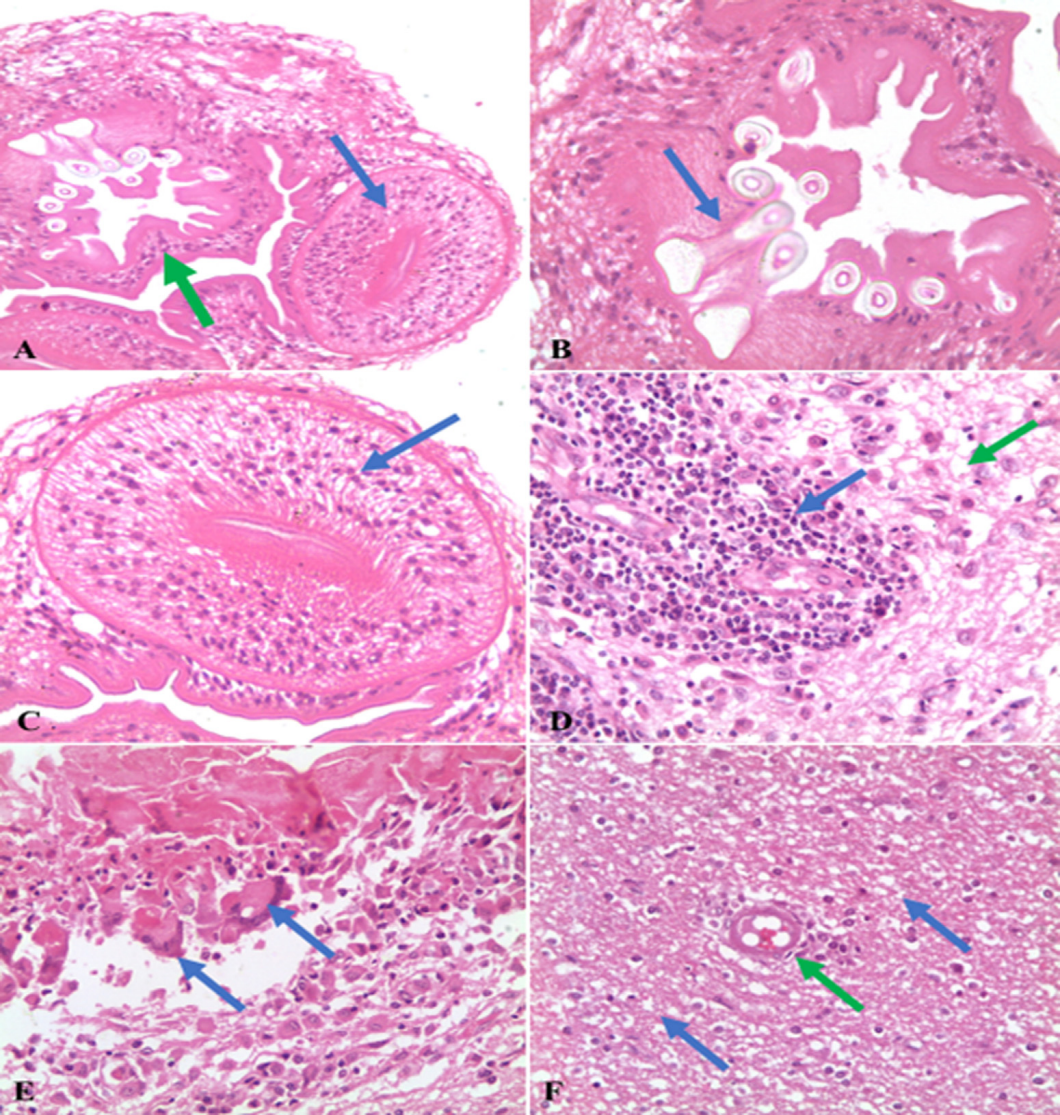
Micrography of brain tissue of sheep infected with *Coenurus cerebralis* larvae stained with hematoxylin and eosin (H&E) showing (A) single scolex of *coenurus cerebralis* with suckers (blue arrow), rostellum (green arrow) and rostellar hocks (blue arrow) [A: X 20, B and C: X 40]. High number of lymphocytes and plasma cells surrounding the veins (blue arrow), there is degenerative changes (green arrow) and demyelination in brain tissue (D: X 20). Multi nucleated giant cells (blue arrow) and macrophages surrounding caseated materials (E: X20). Vacuolation and demyelination (blue arrow) of brain tissue with congested blood vessels (green arrow) (F: X 20).

Pathological data demonstrated atrophy and congestion of cerebral tissue with dilated ventricles. Scolex of *C. cerebralis* larvae appeared embedded in the brain tissue (Fig. 4A, B and C). Congestion of blood vessels, extensive perivascular infiltration with mononuclear inflammatory cells, neural degenerative changes and demyelination in brain tissue (Fig. 4D and F) were prominent features as pathological alterations. Caseated materials due to degenerated cysts were surrounded with multi nucleated giant cells and macrophages (Fig. 4E).

### 1.4 Molecular data

**Fig. 5:**
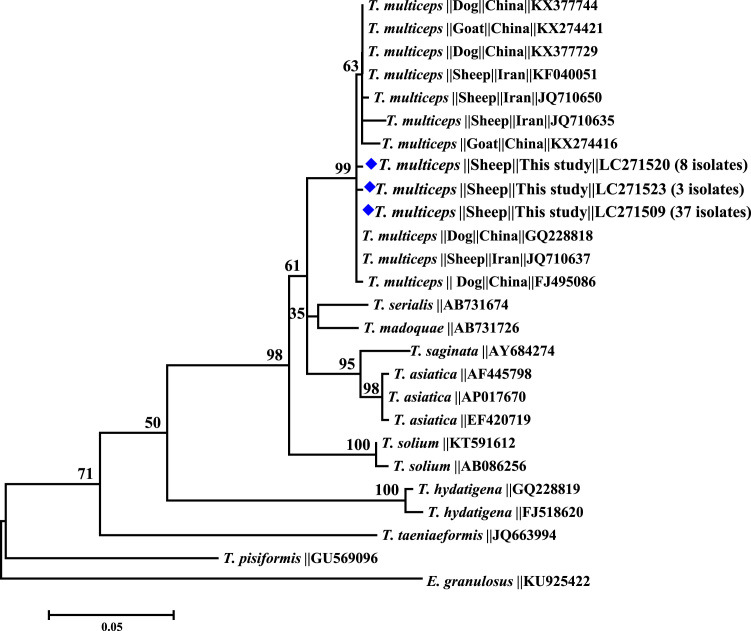
Phylogenetic relationships of *Coenurus cerebralis* from Egypt compared to reference sequences of *Taenia multiceps* in the GenBank database. Evolutionary relationship was inferred based on 12S sequences using the Maximum Likelihood method (ML) as implemented in MEGA6. *Echinococcus granulosus* (KU925422) was used as the outgroup. Sequences obtained in this study are marked with diamonds.

Sequence analysis of the mitochondrial 12S rRNA gene fragment (483 bp) indicated to occurrence of 3 sequences types. The first sequence type was seen in 37 isolates that were identical to the sequence GQ228818 obtained from *T. multiceps* derived from dog in China [2]. The second type of sequences included 8 isolates and showed base transition of A to G at position of 468 compared to the first type, whereas the third sequence type was detected in 3 isolates and showed transition of A to T at position 373 relative to the first sequence type. ML phylogenetic analysis based on 12S rRNA gene sequences clearly showed that *T. multiceps* isolates constitute a mono-phyletic group (Fig. 5). This monophyletic group divides into 2 sister-clades in 12S rRNA ML tree, representing the corresponding variants (Fig. 5). Clustering pattern of *C. cerebralis* isolates in the present study at mitochondrial gene markers revealed clustering of all Egyptian isolates in one cluster for 12S rRNA.

## 2 Materials and methods

### 2.1 Specimen collection

Sheep with clinical signs were brought to local abattoirs at Ashmoun and El Sadat cities, El Menoufia Province (90 km South-East of Cairo, Egypt) for sacrifice. Heads of these animals were physically inspected by veterinary officers at abattoirs for presence of coenurus cysts. Individual cysts were removed from the brain of infected sheep, washed in physiological saline. Cystic fluid from representative number of was obtained (5–32 ml) for biochemical analysis. Protoscolices derived from the cysts were fixed in 10% buffered formalin and mounted on sliders for morphometric measurements. Selected number of cysts (48 cysts) was fixed in 95% ethanol for extraction of genomic DNA. Specimens of brain tissue from infected cases were fixed in buffered formalin solution and prepared for pathological investigation. Formal consent and permission for research use of the cysts were obtained from the attending abattoir veterinarians. No experiments were conducted on live animals.

### 2.2 Biochemical analysis

The fluid from individual cysts (*N*=5) was aspirated with a sterile syringe and centrifuged at 10,000 g at 4 °C for 30 min to remove protoscolices. The supernatant was collected and used to determine glucose concentration [3], total protein [4], urea nitrogen [5], triglycerides, cholesterol, chromium, calcium [6], [7], sodium and potassium [9] values. Aspartate aminotransferase (AST) and alanine aminotransferase (ALT) activities were analyzed by the colorimetric method using commercial kits (Spectrum diagnostics, 30175 Hannover, Germany) according the manufacturer protocol. Data are presented as minimum and maximum values of measurements as well as mean±SD (Table 1).

### 2.3 Pathological investigation

Specimens from representative cases involving cyst and the surrounding tissues were fixed in 10% neutral buffered formalin, washed and dehydrated in graded ethanol, and embedded in paraffin. Five-micrometer-thick sections were stained with hematoxylin and eosin [8] and examined by light microscope.

### 2.4 DNA extraction and PCR analysis

Individual cysts fixed in ethanol were washed extensively with PBS. Genomic DNA was extracted from a small portion of the cyst, contained protoscoleces and/or membrane, using NucleoSpin^®^ Tissue kit (MACHEREY-NAGEL GmbH, Düren, Germany). Mitochondrial 12 S rRNA gene marker was amplified using conditions described by Rostami et al. [9]. PCR reactions were done in 25 μl volume containing 12.5 μl of EmeraldAmp^®^ GT PCR Master Mix (2X premix) [Takara Biotechnology (Dalian) Co., Ltd.], 1 μl of genomic DNA, 0.75 μl (0.3 μM final conc.) of each primer and 10.0 μl of sterile molecular grade H_2_O. No-DNA containing reaction mixture was used as negative control. Each PCR consisted of 30 cycles of denaturation at 98 °C for 10 s, annealing at 57 °C for 35 s, and extension at 72 °C for 50 s. An initial denaturation step at 95 °C for 5 min and a final extension step at 72 °C for 7 min were included. PCR products were visualized by electrophoresis in 1.5% agarose gels.

**Table 1:** Biochemical analysis of *coenurus cerebralis* cystic fluid.

**Sample**	**ALT (U/L)**	**AST (U/L)**	**Triglyceride (mg/dl)**	**Cholesterol (mg/dl)**	**Ca (mg/dl)**	**Cr (mg/dl)**	**Na (mmol/L)**	**K (mmol/L)**	**Glucose (mg/dl)**	**Protein (g/dl)**	**Urea (mg/dl)**
**Minimum value**	42	125	139	145	8.8	1.7	93	4.7	141	5.7	3.3
**Maximum value**	109	165	148	148	11.6	1.8	143	5.9	185	6.3	3.8
**M**±**SD**	69.7±35.0	146.7±20.2	167.7±28.0	147.0±1.8	10.3±1.4	1.8±0.06	125.7±28.3	5.3±0.6	159.3±22.9	6.03±0.31	3.5±0.3

### 2.5 DNA sequence analysis

PCR products were directly sequenced using the Big Dye® Terminator v3.1 Cycle Sequencing Kit and an ABI 3130 Genetic Analyzer (Applied Biosystems, Foster City, CA). Obtained sequences were assembled using the ChromasPro (version 1.5) software (http://www.technelysium.com.au/ChromasPro.html). The accuracy of data was confirmed by bi-directional sequencing. The obtained sequences from each genetic target were aligned with each other and reference sequences using ClustalX (http://www.clustal.org/) to determine the identity of *Taenia* spp. Evolutionary relationship was inferred based the generated sequences using the Maximum Likelihood (ML) method implemented in MEGA7 (http://www.megasoftware.net/). The ML phylogenetic analysis was conducted using the Kimura 2-parameter model and 1000 bootstrap replicates. ML tree was rooted against the nucleotide sequences KU925422 from *Echinococcus granulosus*. Unique sequences from this study were deposited in the GenBank database under accession numbers LC271509 to LC271556.
