# Supplementary material for: Clinical, pathological, and molecular data concerning Coenurus cerebralis in Sheep in Egypt
Source: Data Brief. 2017 Nov 4;16:1–9. doi: 10.1016/j.dib.2017.10.070 (PMC5684426; doi:10.1016/j.dib.2017.10.070)
Supplement: Supplementary file 1 — Supplementary material [file mmc1.doc]

**Conflict of interest**

The authors indicate that there is no conflict of interest in any form in this work
